# Co‐Designing a Framework for Social Media Health Communication to Young People: A Participatory Research Study

**DOI:** 10.1111/hex.70203

**Published:** 2025-03-07

**Authors:** Melody Taba, Julie Ayre, Kirsten McCaffery, Diana Vassilenko, Ivan C. K. Ma, Tara Haynes, Julie Leask, Andrew Wilson, Carissa Bonner

**Affiliations:** ^1^ Sydney Health Literacy Lab, Faculty of Medicine and Health The University of Sydney Sydney Australia; ^2^ Sydney Infectious Diseases Institute The University of Sydney Sydney Australia; ^3^ Leeder Centre for Health Policy, Economics and Data, Faculty of Medicine and Health The University of Sydney Sydney Australia; ^4^ School of Public Health, Faculty of Medicine and Health The University of Sydney Sydney Australia

**Keywords:** co‐design, co‐research, health communication, participatory action research, public health agency, recommendations, social media, young people

## Abstract

**Background:**

Social media became a key communication channel for public health agencies during the COVID‐19 pandemic, especially for reaching younger populations less engaged with traditional channels. However, official social media health communication often fails to appeal to young people. Improving public health agency use of social media for health communication is vital to ensure health messages reach this priority population effectively, especially during public health emergencies.

**Objective:**

This study aimed to co‐design a social media communication framework for health messaging to young people with consideration to emergency settings. It integrated the perspectives of young people and professional stakeholders, health communicators responsible for social media messaging of government health departments.

**Methods:**

An iterative co‐design process was conducted in partnership with youth co‐researchers. The framework was co‐designed over three workshops with young people (18–24 years) and professional stakeholders. Workshop data were analysed collaboratively and the framework was updated iteratively following each workshop. The final framework was approved by the youth co‐researchers and a new group of professional stakeholders.

**Results:**

Twenty‐one young people and four professional stakeholders participated in workshops. Three youth co‐researchers and three external professional stakeholders approved the final framework. Five recommendations for communicating health messages to young people on social media were developed following two iterations: (1) involve young people, (2) pitch at right level, (3) capture attention fast, (4) use current social media marketing techniques and (5) engage more with the public. The main barrier in emergency contexts was time constraints, but the recommendations were considered feasible if embedded in business‐as‐usual processes prior to the emergency.

**Conclusion:**

These findings provide public health agencies a guide for health communication to young people on social media. Co‐designing the recommendations centres the needs and preferences of young people, while ensuring they are feasible for professional stakeholders. By incorporating a variety of messaging approaches and actively involving young people in content development, public health agencies can better reach and engage young people, including during public health emergencies.

**Patient or Public Contribution:**

Young people were involved in study recruitment, workshop facilitation, data analysis and manuscript preparation as co‐researchers. Methods also included co‐design with young people and stakeholders.

## 1 Introduction

Social media has emerged as a major source of health information in recent years, especially during the COVID‐19 pandemic. It became a key channel of health communication for public health agencies, including government health departments, during the early stages of the emergency, given its ability for real‐time messaging and widespread access [1, 2]. It was especially useful to target hard‐to‐reach populations who rarely engage with traditional channels of official health communication, such as young people [3, 4]. Social media effects models suggest that exposure to social media health campaigns can lead to individual behaviour change and improved health outcomes, either directly or indirectly (e.g., exposure through social and policy changes) [5]. Adolescents and young adults are avid users of social media platforms, and access health information on social media very frequently either by actively searching for it on the platforms or passively coming across it on their feeds [6]. Compared with previous generations, Generation Z youth (born 1995–2010) are more likely to use the internet including social media to find out about health‐related topics, whereas earlier generations relied more on traditional media and interpersonal contact [7].

However, young people frequently face health literacy challenges when navigating online health information, in part due to their limited experience with the health system and the lack of tailored, age‐appropriate health information from public health agencies [8, 9]. Specifically, young people report difficulty finding credible health information online and often overestimate their digital health appraisal skills [10, 11]. This may leave them vulnerable to online health misinformation, false information spread without the intention to mislead [12] which proliferates on social media platforms [13]. Furthermore, misinformation is often spread by influential online figures, skilled at presenting engaging content on social media in ways that appeal to young people. For example, a recent study found that ten ‘super spreader’ social media accounts were responsible for originating over 34% of the misinformation on the platform [14]. This influence may further be amplified during health crises which can be accompanied by a flood of incomplete, false or misleading information, as seen during the COVID‐19 pandemic [15].

To combat this, public health agencies must actively fill information voids online with reliable information that resonates with young people to prevent misinformation from dominating [16]. However, whilst public health agencies have generally increased their social media presence, more so during the COVID‐19 pandemic, their messaging is often unappealing to young people, and posted on platforms they do not use [17, 18]. Currently, there are limited frameworks that support health agencies in social media health communication, and even fewer focussing on young people. The literature available thus far is limited to perspective pieces without empirical data [19, 20, 21]. Furthermore, many of the health agencies' social media guidelines are out of date and do not include newer platforms like TikTok (e.g., Centers for Disease Control and Prevention (CDC)'s 2011 Social Media Toolkit [22] or European Centre for Disease Prevention and Control (ECDC)'s 2016 Social Media Strategy Development [23]) or are limited to a certain health topic (e.g., World Health Organization (WHO)'s COVID‐19 Misinformation Toolkit [24]). Whilst there has been some research in social media crisis communication emerging in recent years [25], a scoping review has found a general insufficiency of social media guidance policies for public health agencies' specifically around emergencies [1]. For example, prominent frameworks for emergency communication (e.g., WHO's Guideline for Emergency Risk Communication [26]) have limited social media‐specific guidance.

Such frameworks can offer health communicators a clear, structured guide to follow to ensure their messaging and outreach efforts are consistently effective. Co‐designing a framework is a potential solution, a process in which active collaboration occurs between the stakeholders (young people, public health agencies and researchers) in designing solutions to a prespecified problem [27]. Co‐designing such a framework ensures outputs are relevant to young people's needs and experiences, as well as supporting future implementation by health agencies [28]. Co‐design approaches are also suitable for young participants since they can utilise creative and interactive activities which can make young people feel more comfortable when discussing their experiences in traditional research settings [29].

## 2 Aim

This study aimed to co‐design a framework to help public health agencies use social media more effectively for health communication to young people. It brings together the perspectives of young people and health communicators in government health departments who are responsible for social media messaging (professional stakeholders).

To achieve this, we had the following objectives:

1. Identify young people's perspectives and preferences on social media health communication from public health agencies.
2. Identify the feasibility of these preferences and relevant emergency considerations with professional stakeholders at public health agencies.

## 3 Methods

### 3.1 Study Design

We used a qualitative approach to co‐design the framework with young people and health communicators specialising in social media. The process included three workshops and subsequent analysis to develop, refine and finalise the framework iteratively.

We engaged in participatory action research methods [30], beyond the co‐design workshops and involved young people as co‐researchers to strengthen the research process and improve the effectiveness and relevance of outputs to young people as outlined in the WH&Y Youth Engagement in Health Research Framework [31].

Ethics approval was obtained from the University of Sydney Human Research Ethics Committee (2022/887).

### 3.2 Study Team

Three youth co‐researchers (D. V., T. H., I. M.) were engaged throughout the study as part of the research team alongside health policy and health communication researchers with diverse experiences and seniority. Co‐researchers were aged 21–24 years, identified as man or woman and had diverse cultural backgrounds (Hong Kong, Kazakhstan and UK). Two were trained research assistants working with senior members of research team on other projects and one was a consumer trained in the required research methods for this study. One co‐researcher also had experience as a social media content creator (67,000 followers across Instagram, TikTok and YouTube). Co‐researchers were organised by PhD candidate (M.T.; woman, aged 28, Iranian background) who served as a liaison to reduce power imbalance and potential social desirability bias between young co‐researchers and senior researchers.

The youth co‐researchers were involved throughout the research process from recruitment to data collection and analysis. Their contributions included developing and piloting workshop activities, designing recruitment flyers to ensure engagement from young people, co‐facilitation of the workshops, collection of field notes and synthetises of ideas raised at the workshops. They also led the set‐up of the in‐person workshop venues to ensure they were comfortable for young participants, selecting catering, background music, lighting and setting up a charging station to ensure participants' phones were charged.

### 3.3 Participants

There were two stakeholder groups in the co‐design process: professional health communicators and young people. Whilst the framework was intended primarily for professional stakeholder use, it was ultimately designed with the needs and preferences of young people at its core to achieve the greatest benefit to them.

The professional stakeholders were health communicators with experience working in the social media team of Australian government state/jurisdiction health departments. They were purposively sampled from a pool of previously identified stakeholders for a related interview study (to be reported separately) and were recruited via a study invitation email. All invited stakeholders were involved in the official government COVID‐19 social media emergency response in a range of roles (i.e., manager or content creator).

The young people were aged 18–24 years and were recruited via flyers advertised on social media. Purposive sampling (maximum variation sampling) was used to recruit diverse participants with a range of ages, genders and social media usage to enhance the diversity of perspectives obtained and ensure broad relevance of the framework.

### 3.4 Co‐Design Workshops

**Figure 1:**
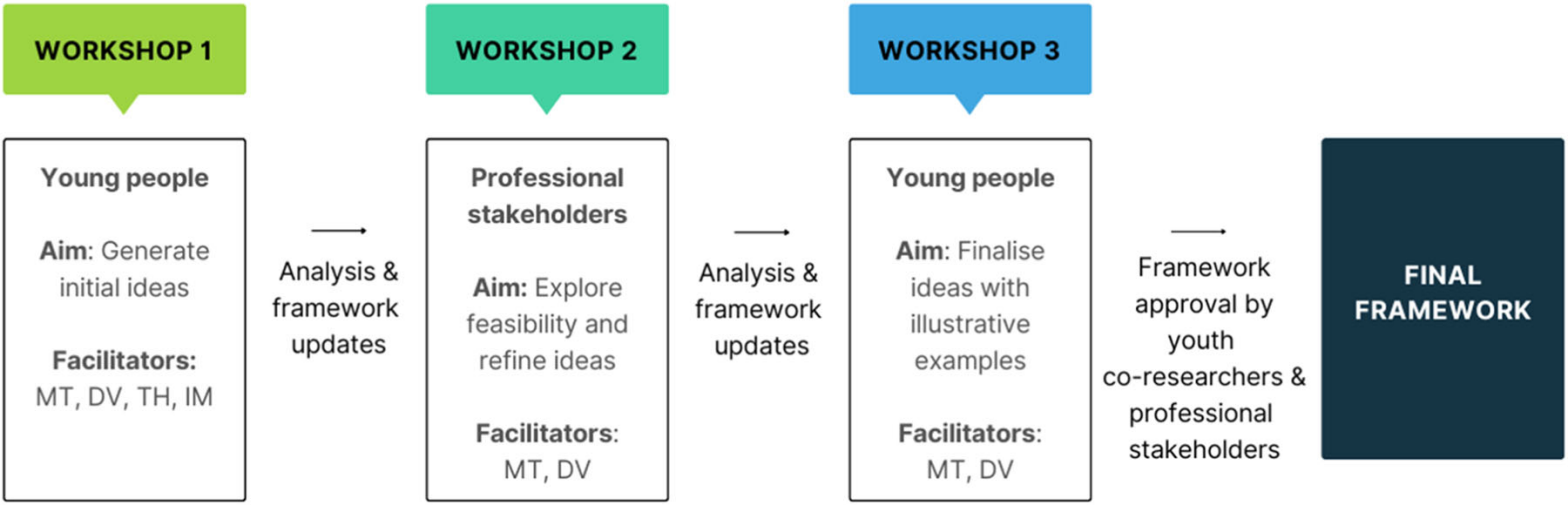
Co‐design process overview.

Three co‐design workshops were conducted between October 2023 and March 2024 (Figure 1). Workshop 1 was conducted with young people and focussed on generating initial ideas for the framework. Workshop 2 was conducted with professional stakeholders and focussed on the feasibility and refinement of young people's ideas. Workshop 3 was conducted with young people and focussed on finalising the recommendations in the framework with illustrative examples.

The workshops with young people were conducted in‐person over 2 h, and the workshop with the professional stakeholders was conducted online over 1 h using Zoom teleconferencing. Workshops were facilitated by M. T. and youth co‐researchers, D. V., T. H. and I. M. All participants were offered a $100 gift card to acknowledge their contributions to the study.

### 3.5 Workshop Activities and Data Collection

The workshops included a range of data‐generating activities like finding and assessing examples of official social media health messaging and live graphic illustrations capturing key ideas, as well as World Café style [32] and traditional focus group discussions. Activities and resultant artefacts (tangible and shareable outputs of the research and design process [33]) are described in Table 1. Physical and digital artefacts generated in the workshops included annotated tables and affinity diagrams (visual brainstorms that allowed participants to organise ideas according to their natural relationships [34]). Example artefacts in Supporting Information S1.

**Table 1:** Activities conducted in study workshops and subsequent artefacts generated.

Workshop details	Activity	Artefacts generated
Workshop 1 Young people	**Assess & rank the posts**: participants assessed and ranked social media posts from public health agencies in small groups on a digital board with youth facilitator.	Annotated table
	**Search for posts**: participants individually searched their own social media feeds for health information posts which were discussed in small groups to identify aspects that were liked and disliked. Youth facilitators organised ideas onto a digital board.	Annotated table
	**World Café [** 32 **]**: participants rotated in small groups through multiple rounds of focus groups on the following topics: (a) what makes health‐related social media posts appealing, (b) what should health agencies do more/less of on social media and (c) recommendations for health agencies to improve their social media messaging. Youth facilitators captured key ideas on a digital board with sticky notes before categorising and collating by themes.	Affinity diagram
	**Live graphic recording**: a professional illustrator recorded key ideas raised throughout the whole workshop. The illustration was displayed to participants throughout the workshop, and they gave feedback to the illustrator to incorporate into the final graphic recording in real time.	Professional illustration
Workshop 2 Professional stakeholders	**Barriers and enablers**: participants were shown the framework developed after Workshop 1 and discussed the feasibility of each recommendation, identifying enablers and barriers. Youth facilitator captured key ideas on a digital table.	Annotated table
	**Focus group**: participants discussed the following topics as group: (a) how can the framework be refined, (b) how can the framework be made systematic and (c) how would the framework change in emergencies. Youth facilitators captured key ideas on a digital board with sticky notes before categorising and collating by themes.	Affinity diagram
Workshop 3 Young people	**Sort the posts**: participants were shown new social media posts from public health agencies and identified if any were illustrative examples of the recommendations in the framework. Participants sorted the posts into the relevant recommendation on a physical board with sticky note annotations.	Affinity diagram
	**Focus group**: participants discussed any final feedback they had for the recommendations. Participants added further sticky note annotations to the physical board from the previous activity.	Affinity diagram

Field notes were taken by all facilitators and the workshops were audio‐recorded and transcribed.

### 3.6 Data Analysis

Data analysis was informed by the participatory action research approach of collaborative analysis [30]. Initial data analysis occurred during workshop activities since generating artefacts required idea synthesis [30]. For example, participants and youth facilitators organised ideas into categories to generate affinity diagrams and the professional illustrator summarised ideas during graphic recording, amending the graphic immediately based on participant feedback during the workshop.

Upon completion of the first workshop, M. T., D. V., T. H. and I. M. digitised and further collaboratively analysed the data by reviewing the contents of the artefacts, identifying and categorising concepts into an initial concept map using the web application Miro. Field notes and workshop transcripts (transcribed by external company) were also reviewed to ensure key concepts were not missed in analysis. The broader study team including J. A., K. M. and C. B. updated the concept map with subsequent iterations following each workshop based on participant feedback, further refining in weekly meetings until the final recommendations were developed. Youth co‐researchers and three professional stakeholders who did not participate in the workshops but were interviewed for a related study reviewed and approved the final recommendations.

## 4 Results

A total of 21 young people participated across the two youth workshops (Workshop 1: *n* = 14, Workshop 3: *n* = 7) (Table 2). The mean age was 20.2 years, 53% identified as men, 43% as women and 4% as other gender (*n* = 1). All used the social media platform Instagram, 62% used Facebook and 48% used TikTok. The majority (81%) were studying or had studied at university.

Four professional stakeholders participated in the stakeholder workshop. All stakeholders were women in their 30s; half (*n* = 2) were aged 30–34, reflecting typical workforce characteristics. They represented a range of roles in the communication department of an Australian health department, ranging from social media officer to the communication director, experience ranging between 5 and 15 years.

**Table 2:** Workshop participant demographics.

Young people (Workshops 1 & 3)	*N* (total = 21)
Age	
18–29	11
21–24	10
Gender	
Man	11
Woman	9
Other	1
Social media used	
Instagram	21
Facebook	12
TikTok	10
Twitter/X	7
Youtube	6
Occupation	
Student	17
Other	4
**Stakeholders (Workshop 2)**	***N* (total = 4)**
Age	
30–34	2
35–39	2
Gender	
Woman	4
Occupation	
Communication Director	1
Social Media Manager	2
Social Media Officer	1
Years of experience in role	
5–10 years	2
10–15 years	2

**Figure 2:**
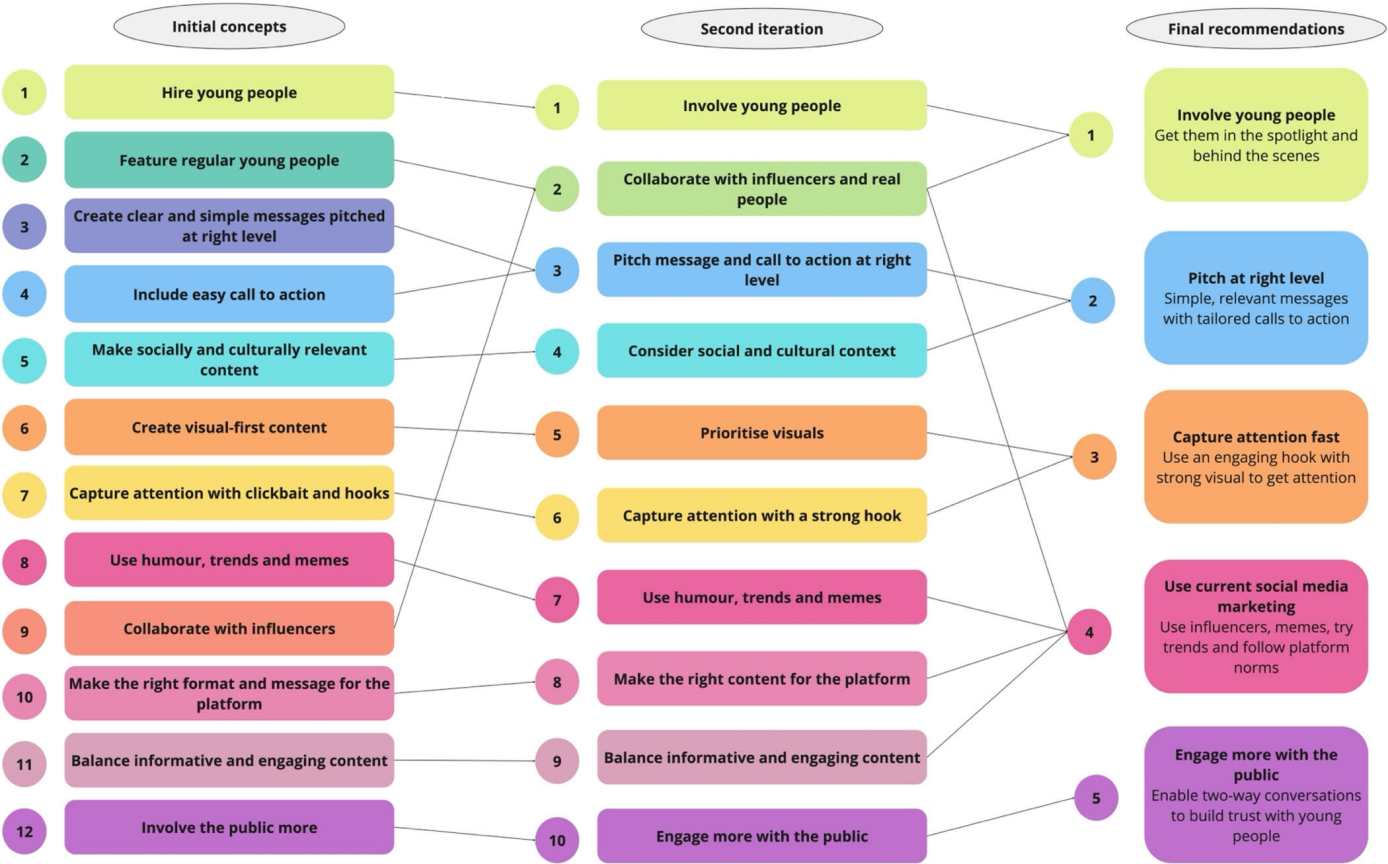
Iterations of framework leading to final recommendations for social media health communication to young people.

**Figure 3:**
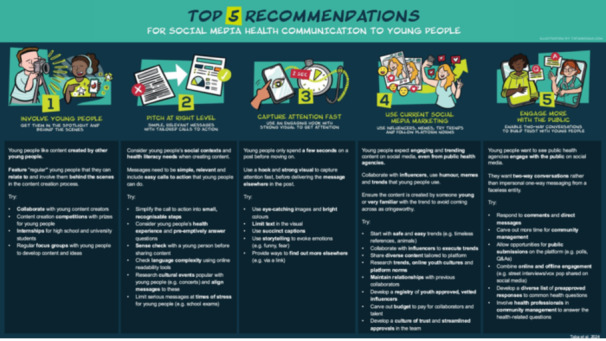
Final co‐designed recommendations with illustrative examples. *Source:* Originally published at Sydney eScholarship Repository [35].

The iterative co‐design process resulted in five final recommendations for communicating health messages to young people via social media; (1) involve young people, (2) pitch at right level, (3) capture attention fast, (4) use current social media marketing, (5) engage more with the public. Early iterations are outlined in Figure 2, and findings from each individual workshop are outlined in Supporting Information S2 and final framework with illustrative examples outlined in Figure 3.

### 4.1 Recommendation 1: Involve Young People

Young people reported a preference for social media content featuring, and created by, their peers. They found this content more relevant and relatable, and believed young people are better placed to communicate message to their peers. Initially, young people recommended they should be hired specifically in social media roles as explained by one participant:

‘If they're targeting young people, they should hire young people around of our age to make the posts so it is always relevant to us’.

Young person, Workshop 1

Whilst stakeholders reported barriers to directly hiring young people (e.g., age discrimination policies, time and budget constraints for creating new roles), they explained that often their staff are in the younger age category already:

‘You will generally get a younger group of people working in social media roles, like in my team we have a 22 year old. They are always on top of the trends. They usually the ones creating content. That's the nature of social media, you tend to get younger people entering the space’.

Stakeholder, Workshop 2

The stakeholders suggested young people could also be involved in other ways for example, via internships and short‐term positions that provide practical experience for recent graduates:

‘Cadetships for people coming out of school or in university…In doing that you upskill them into the workforce but you also have the benefit of leveraging them for the demographic you're trying to target’.

Stakeholder, Workshop 2

Stakeholders also recommended organising collaborations with young content creators for proactive campaigns and regularly scheduled focus groups with young people to brainstorm and sense‐check content with young people. Young people agreed with these suggestions in the subsequent workshop upon hearing of the stakeholder limitations. They suggested that public health agencies could also involve young people by organising content creation competitions on social media and showcase winners that aligned with organisational values.

‘They should start competitions where they encourage young people to make an informative video on some health issue for social media…with a financial incentive’.

Young person, Workshop 3

The professional stakeholders considered involving young people was less feasible during emergencies due to limited time to train and organise opportunities. However, they reported that if young people were embedded in the system prior to the emergency (either as interns, or accessed via regularly scheduled focus groups), it would ensure they were involved in emergency communications with minimal changes to usual process.

‘You want seasoned people in a crisis. You can't afford to spend much time coaching people but that's part of the preparedness – if you have young people part of the team, they will be ready to go when the time comes’.

Stakeholder, Workshop 2

### 4.2 Recommendation 2: Pitch at Right Level

Young people wanted health messaging that was tailored and pitched to their age group on social media. Specifically, they wanted simple health information that considers their lack of experience with the health system, free from complex language and medical jargon. As one participant explained:

‘Give us bite‐sized information instead of being a whole burger’.

Young person, Workshop 1

Young people also reported they were unsure what they were ‘meant to do’ after seeing a health message on social media. They wanted straightforward calls to action which anticipated and clearly explained all required actions. Many were unfamiliar with the health system or had not made health decisions before so needed extra instructions around what to expect.

‘I need more than just “get vaccinated”, tell me exactly what steps I need to take to get it’.

Young person, Workshop 1

They also recommended that health agencies consider young people's social and cultural contexts in their messaging, for example, aligning messaging with youth events and avoiding serious health messages during times of high stress for the age group (e.g., school exams).

‘Young people like concerts and events so use those trending concerts to get information across’.

Young person, Workshop 3

The professional stakeholders agreed with this recommendation, explaining messaging could be simplified and sense‐checked by young people before sharing, and cultural contexts can be researched to align messaging.

‘I'm obviously not going to be across all of the different social issues and cultural norms. That's where you need to do some research, but also having different advisory panels would be helpful in those situations’.

Stakeholder, Workshop 2

‘We can maybe strip language down a bit. It's hard because there are lots of tricky words in health’.

Stakeholder, Workshop 2

Stakeholders considered this recommendation could be feasible during emergencies but that sense‐checking and research opportunities are lost when messages need to be disseminated immediately. As above, they reported that if young people are already embedded in the system prior to the emergency, more tailored and age‐appropriate messaging could always be enabled.

### 4.3 Recommendation 3: Capture Attention Fast

Young people reported they only spend a few seconds on an individual post ‘before scrolling away’ on social media, so messaging must capture their attention immediately. They liked strong, eye‐catching, visuals with minimal text and short but informative caption, as one participant explained:

‘If we're talking about Instagram or Facebook, the number one thing that catches my attention is like clean, simple graphics with good line spacing in the caption’.

Young person, Workshop 1

They also favoured content with a hook or compelling element to grab attention in the initial part of a post to encourage further engagement with the message.

‘You need some kind of click‐bait first, then you have to incorporate a story throughout the post and add a link with more information’.

Young person, Workshop 1

Whilst professional stakeholders agreed with incorporating strong visuals and a hook in their messaging, they were against the term ‘clickbait’ since misleading content can erode trust for health agencies:

‘Hook is probably more appropriate, click‐bait is a negative term for us’.

Stakeholder, Workshop 2

The young people at the subsequent workshop agreed with this updated recommendation. They suggested an example of capturing attention with a strong hook may include an initial evocative image, which compelled them to read a short but informative caption before being encouraged to research the topic more via a link in the post.

‘Strong hooks in the beginning of a health post would keep me interested, even during the boring stats part’.

Young person, Workshop 3

‘I think the post on social media should be funny and engaging, but then the link should have more information and be serious. If it's a serious post straight away, I think a lot of people will scroll past it…’.

Young person, Workshop 3

The stakeholders considered this recommendation as feasible during emergencies, yet less relevant due to heightened anxiety and interest around health issues.

### 4.4 Recommendation 4: Use Current Social Media Marketing

Young people reported they expect engaging and trending content on social media, even from government health agencies. Specifically, they recommended packaging health messages in the context of social media trends, humorous memes, popular influencer collaborations and diverse formats.

‘The best way to reach our generation on social media is to go along with the trends while still highlighting the main information that the government wants to get across’.

Young person, Workshop 1

They reported they were more likely to engage and share this type of content with their friends, but only if the execution was on trend, and the content seemed authentic and genuine if coming from an influencer. They recommended influencers or young people are well suited to create this type of content in collaboration with health agencies to ensure effective execution of trends.

‘Stay away from cringey stuff. It's looked down upon. Some trends are viral because it's bad. That's not what you want to do’.

Young person, Workshop 1

‘You can use memes as long as it's something that's appealing to the young generation. So not having someone too old to make the posts themselves because we can tell you're not on trend’.

Young person, Workshop 1

‘It's important to use the influencers well. Like make it more authentic, otherwise everyone thinks they are selling out or they got paid to do it. If you're gonna work with an influencer, give them creative control because we can tell when it's a script’.

Young person, Workshop 3

Professional stakeholders reported multiple barriers to using social media marketing strategies including cost and difficult approval processes. However, feasibility could improve as senior management increasingly recognise the importance of social media marketing:

‘The thing with trends is we have long, lengthy approval process and then you're posting about something that was two months ago. But it won't be as relevant and then it comes across as dated to a young person’.

Stakeholder, Workshop 2

‘A lot of our issues are at the top. If people at the top aren't willing to approve, then we can't do much. But we can build trust with our executive and show them how it works’.

Stakeholder, Workshop 2

They further recommended that a registry of vetted influencers could be developed for proactive campaigns and budgets to be carved out specially for these collaborations. Young people in the subsequent workshop also suggested some ‘safe’ trends for health agencies including timeless references (e.g., classic film references or content featuring cute animals).

This recommendation was considered less feasible during emergencies by the stakeholders due to time constraints and sensitivity required for managing an emergency:

‘It's harder to be funny when there is a serious emergency’.

Stakeholder, Workshop 2

Preparation would be required such as maintaining relationships with previous collaborators or developing a registry of vetted influencers. Again, the need to engage in social media marketing was considered less relevant during an emergency due to heightened anxiety and interest around health issues.

### 4.5 Recommendation 5: Engage More With the Public

Young people recommended health agency engage in two‐way conversations with the public on social media. This included replying to messages individually and responding to user comments, particularly in an interesting and entertaining way. They explained seeing this type of interaction would engender trust and foster healthy discussion about important health topics.

‘I like when they [public health agencies] respond to comments in a tongue‐in‐cheek way and shut down the misinformation trolls’.

Young person, Workshop 1

‘It makes them seem less like an entity and more like a real person’.

Young person, Workshop 3

‘There is such a large population who have distrust for the government so it's good to have healthy and open discussions. Like social media is supposed to be a place where the public can have these discussions with those officials so they can understand and learn about what they do’.

Young person, Workshop 3

Whilst some of these suggestions faced approval and reputational risk barriers, the stakeholders recognised the value of community engagement.

‘Community management is one of the best ways to build trust with your audience. You can directly combat misinformation and it helps with the algorithm’.

Stakeholder, Workshop 2

They suggested greater time should be dedicated to community engagement and related activities, such as developing a diverse and comprehensive list of preapproved responses to allow for more comment responses and allowing opportunities for public submission (e.g., health questions or topics of interest) via platform features (e.g., Instagram Polls):

‘Yeah, it's definitely something we're conscious of doing, like quizzes or getting questions in those functions on Instagram. It helps us as well because we hear what they want to see’.

Stakeholder, Workshop 2

Professional stakeholders reported this recommendation was less feasible during emergencies due to time constraints and shift in focus to on one‐way message dissemination. However, they also noted the increasing importance of community engagement during these contexts since the public are generally more anxious and have more questions for health agencies.

## 5 Discussion

### 5.1 Principal Results

This study co‐designed five key recommendations for public health agencies to use when communicating health messages to young people on social media; (1) involve young people, (2) pitch at the right level, (3) capture attention fast, (4) use current social media marketing and (5) engage more with the public. In general, the recommendations were considered feasible during emergencies if embedded in business‐as‐usual processes prior to the emergency. Some recommendations (capture attention fast and use social media marketing) were viewed as less essential during emergencies due to heightened public anxiety and increased interest in health messages. However, the recommendation of engaging with the public would become more important during times of uncertainty.

Like other co‐design studies working with young people and professional stakeholder groups, the framework had to strike the right balance between the preferences of young people and the constraints of the health agencies [36, 37]. There were several ideas young people raised that were simply not possible for the professional stakeholders (e.g., sharing click‐bait or ‘shutting down misinformation trolls’ in the comments). However, many of these ideas could be revised to reach a middle ground idea that was acceptable to both stakeholders (e.g., alternative hooks or constructive comment responses to misinformation).

### 5.2 Comparison With Prior Work

This study supports principles recommended in previous research on social media health communication to youth such as collaborating with young people as messengers, using age‐appropriate language, and considering young people's interests in health messaging, outlined by Sobowale and colleagues [21]. This framework also shares elements with social media health communication frameworks designed for general populations. For example, de Vere Hunt and Linos [19] outlined the importance of involving members of target population in message development, tailoring of social media messaging to specific populations and leveraging shareability with social marketing strategies. Gatewood and colleagues [20] further recommended understanding and using appropriate platforms and consistently creating content that engaged audience using high visual images. A scoping review identified similar guidelines for public health agencies' use of social media during emergency contexts, including fostering public interaction and engagement, leveraging platform features, considering needs of message recipient and recognising members of the public as partners in co‐creating and disseminating messaging [1]. Other principles are also echoed in official emergency communication guidance with WHO, CDC, ECDC and Canadian Public Health Association recommending involving consumers, simple and empathetic messaging, including calls to action and building trust with the public in their health emergency response frameworks [22, 23, 24, 38].

The proposed framework is unique in that it applies such principles to the specific needs of young people in the current social media context (i.e., considering newer platforms young people now use like TikTok). The framework also provides practical examples to guide health agencies in applying the recommendations in emergency and non‐emergency phases. Social media is crowded with competing voices and information sources, which means health agencies cannot just assume any of their messages will automatically capture and hold the attention of its users, especially considering competitors are often very skilled at creating engaging content [39]. This is especially pertinent in the context of young audiences, who have unique social media cultures and norms [40]. As seen in our findings, young people have different needs when it comes to social media health communication. This includes both health literacy‐related considerations, as young people need simple messaging with limited jargon, but also other age‐related considerations including youth cultures and youth social media cultures [8, 21, 40]. These issues can be addressed by involving young people in the communications to ensure the messaging is appropriate for young people every time.

### 5.3 Practical Implications

These findings provide a framework with practical examples to guide public health agencies in using social media to reach young people. This framework can also be considered in circumstances when government agencies outsource communications to commercial firms. While participants raised some feasibility issues during emergencies, the framework is flexible, and the recommendations can be used in isolation or combination as appropriate to the context.

As seen in our findings and previous research, it is imperative to leverage two‐way communication on social media to build trust and engagement with health messages [5]. Audiences are not passive information receivers on social media, but active participants who can interact with campaign messages, amplify reach and influence the wider online communities' perspectives contributing to message acceptance, uptake and trust [41, 42, 43]. Platforms also easily allow for monitoring of audience sentiment via ‘social listening’, as well as immediate responses to audience questions [44]. This is incredibly important during major health crises as fostering public engagement and trust with health agencies becomes more important whilst also providing them a platform for combatting misinformation effectively [1, 25, 45]. For example, research has found that rebuttal of COVID‐19 vaccine misinformation by public health agencies on social media reduced the public's belief in the misinformation and increased their willingness to get a COVID‐19 vaccine [43]. This is an appealing feature to young people, who are less interested in one‐way didactic messaging, and prefer (and even expect) the two‐way communication as seen in our findings. Young people may also observe these public interactions ‘silently’ without publicly engaging, but these interactions can still influence their attitudes towards health messages [46].

Using social marketing was another unique affordance to be leveraged for health messaging on social media, as highlighted in our study and previous research [21, 47, 48, 49, 50]. For example, partnering with influencers, as recommended in our framework, has been successful in influencing social norms and health behaviours related to COVID‐19 and influenza vaccination [47, 50]. Organisations like WHO have already implemented this, creating a registry of vetted influencers via the “Fides” programme, which health agencies could use as a model to develop their own local equivalents [51]. As seen in our findings, social media collaborations must be authentic and allow the influencer's own interpretation of the campaign to better speak to the audience for optimum effect [50]. However, as explained by the stakeholders in our study, such collaborations may be challenging to execute during emergency situations and may be risky for health agencies. Public health‐related posts may contrast with usual influencer content and undermine content coherence, a key factor for social media influencer effectiveness [52]. This issue warrants further research on how trusted partnerships between health agencies and influencers could be built over time, in preparation for future emergencies. Different approaches may be needed on different social media platforms, such as TikTok versus Twitch [53, 54].

### 5.4 Strengths and Limitations

The key strength of the framework lies in its collaborative development. Bringing together the perspectives of young people and health communicators through co‐design improves its acceptability and feasibility for all stakeholders, at multiple levels. The participatory approach involving youth co‐researchers also strengthened the study. Involvement of co‐researchers improved participant experience, and thus quality of the data generated, as well as introducing new ideas to study team about youth‐centred research practice (i.e., importance of workshop ambiance and phone charging stations for young people).

A limitation due to convenience sampling meant there were only a small number of professional stakeholder participants, and they were all affiliated with the same Australian health department. Stakeholders from other organisations may operate under different processes which may have impacted final recommendations. However, all study stakeholders had diverse roles within their organisation and had experience working for other government organisations. The final framework was also checked by a larger and more diverse group of professional stakeholders from other health departments who were involved in a related study [18] to ensure the recommendations are appropriate for a range of organisations. Another limitation is that most participants were university students, whose health messaging needs and preferences may differ from non‐students. Finally, the recommendations reflect the social media preferences that young people expressed during the workshops but may not translate to their actual engagement on social media. Future research could consider methods involving citizen science (i.e., data donation) and tracking a diverse group of young people to explore their actual engagement with social media content from public health agencies [55].

### 5.5 Future Directions

We recommend this co‐designed framework is implemented for future health communication to young people in advance of emergency conditions as part of preparedness efforts. Whilst the recommendations were specifically developed with professional health communicators at Australian government health departments, the findings will be relevant to any organisation communicating health messages to young people via social media. Successful implementation of the framework ultimately requires investment, resourcing and buy‐in from senior management [21]. Furthermore, social media campaigns should be evaluated to understand the real‐world outcomes [19, 20]. Though social media is constantly evolving, the underpinning principles of this framework of youth engagement and leveraging new technologies for health communication will continue to be important as the landscape develops. Whilst this work aims to reduce the health literacy demands of the online information environment for young people, it must be paired with health literacy education and appropriate referral to health professionals to prevent issues like misinterpretation and self‐diagnosis.

## 6 Conclusion

This research provides a practical, co‐designed framework to guide public health agencies using social media to reach young people, to ensure access to reliable yet appealing health information. Co‐designing the recommendations centres the needs and preferences of young people, while ensuring they are feasible for professional health communicators. By incorporating a variety of messaging approaches and actively involving young people in content development, health agencies can better reach, engage and impact the health behaviours of young people. Capacity for this messaging needs to be built in business‐as‐usual times as part of preparedness efforts so that health agencies can provide high‐quality, youth‐centred social media communications effectively during future public health emergencies.

## Author Contributions

**Melody Taba:** conceptualisation, investigation, funding acquisition, writing – original draft, methodology, visualisation, writing – review and editing, formal analysis, project administration. **Julie Ayre:** conceptualisation, investigation, funding acquisition, methodology, writing – review and editing, supervision, formal analysis. **Kirsten McCaffery:** conceptualisation, investigation, funding acquisition, methodology, writing – review and editing, supervision, formal analysis. **Diana Vassilenko:** investigation, writing – review and editing, formal analysis, methodology. **Ivan C. K. Ma:** investigation, methodology, writing – review and editing, formal analysis. **Tara Haynes:** investigation, methodology, writing – review and editing, formal analysis. **Julie Leask:** conceptualisation, funding acquisition, writing – review and editing, supervision. **Andrew Wilson:** conceptualisation, funding acquisition, writing – review and editing, supervision. **Carissa Bonner:** conceptualisation, investigation, funding acquisition, methodology, writing – review and editing, formal analysis, supervision. All authors reviewed the results and approved the final version of the manuscript.

## Ethics Statement

Ethics approval was obtained from the University of Sydney Human Research Ethics Committee (Approval Number 2022/887). Figure 3 is originally published by authors in the Sydney eScholarship Repository under CC BY 4.0. Permission is granted to reproduce material and appropriately attributed and cited in this manuscript.

## Conflicts of Interest

The authors declare no conflicts of interest.

## Supporting information

Supporting information.

Supporting information.

## Data Availability

The data that support the findings of this study are available in the Supporting material of this article, otherwise available on request from the corresponding author. Some data are not publicly available due to privacy or ethical restrictions.
