# Supplementary material for: Co‐Designing a Framework for Social Media Health Communication to Young People: A Participatory Research Study
Source: Health Expect. 2025 Mar 7;28(2):e70203. doi: 10.1111/hex.70203 (PMC11886886; doi:10.1111/hex.70203)

# **APPENDIX 1: EXAMPLES OF ARTEFACTS GENERATED FROM WORKSHOP**

# Ranked list (a), affinity diagrams (b & c).


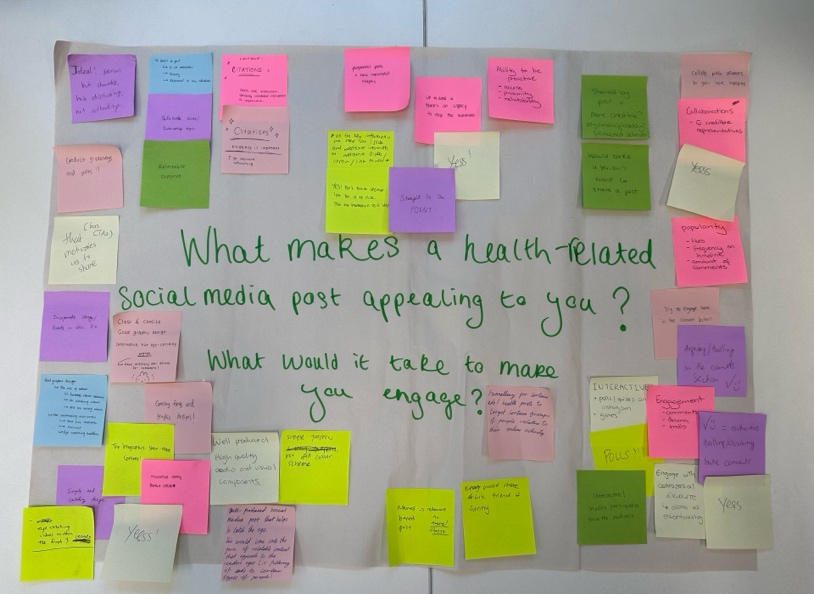

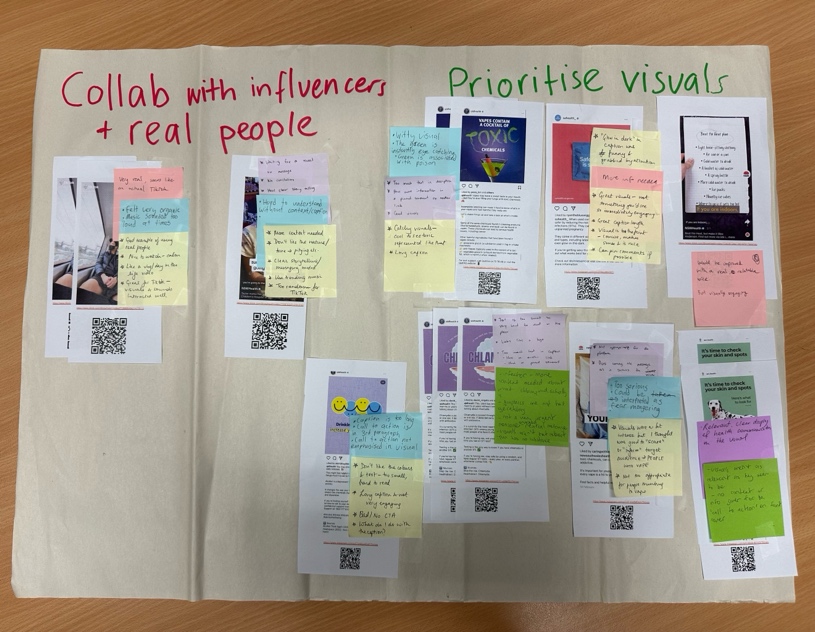


**a**

**b**

**c**


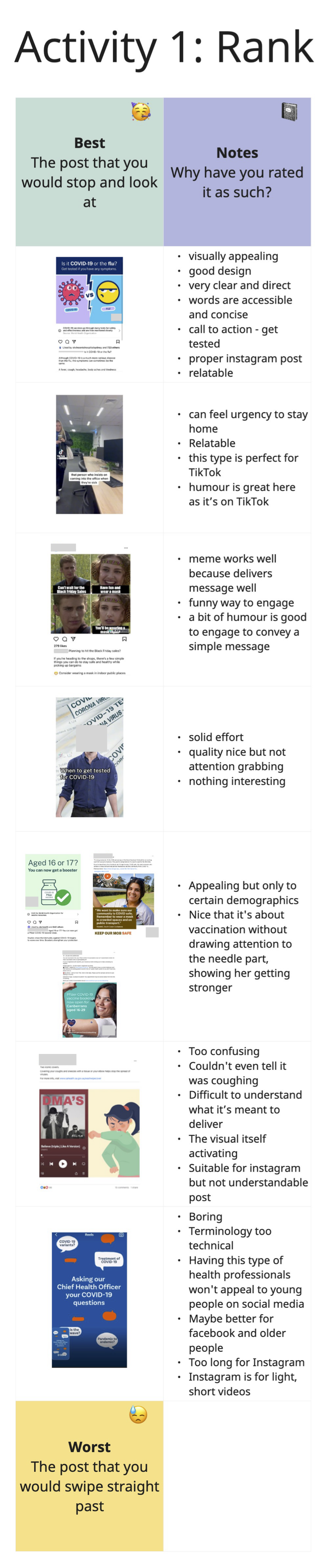

Supplement: Supplementary file 1 — Supporting information. [file HEX-28-e70203-s002.docx]
