# Supplementary material for: Co‐Designing a Framework for Social Media Health Communication to Young People: A Participatory Research Study
Source: Health Expect. 2025 Mar 7;28(2):e70203. doi: 10.1111/hex.70203 (PMC11886886; doi:10.1111/hex.70203)
Supplement: Supplementary file 2 — Supporting information. [file HEX-28-e70203-s001.docx]

# **APPENDIX 2: FINDINGS FROM EACH WORKSHOP**

**Workshop 1 Findings**

Young people shared their preferences for official social media health messaging and their initial recommendations for public health agencies in Workshop 1 (Figure A, Box A)

**Figure A**: Graphic recording of young people’s ideas during Workshop 1

***
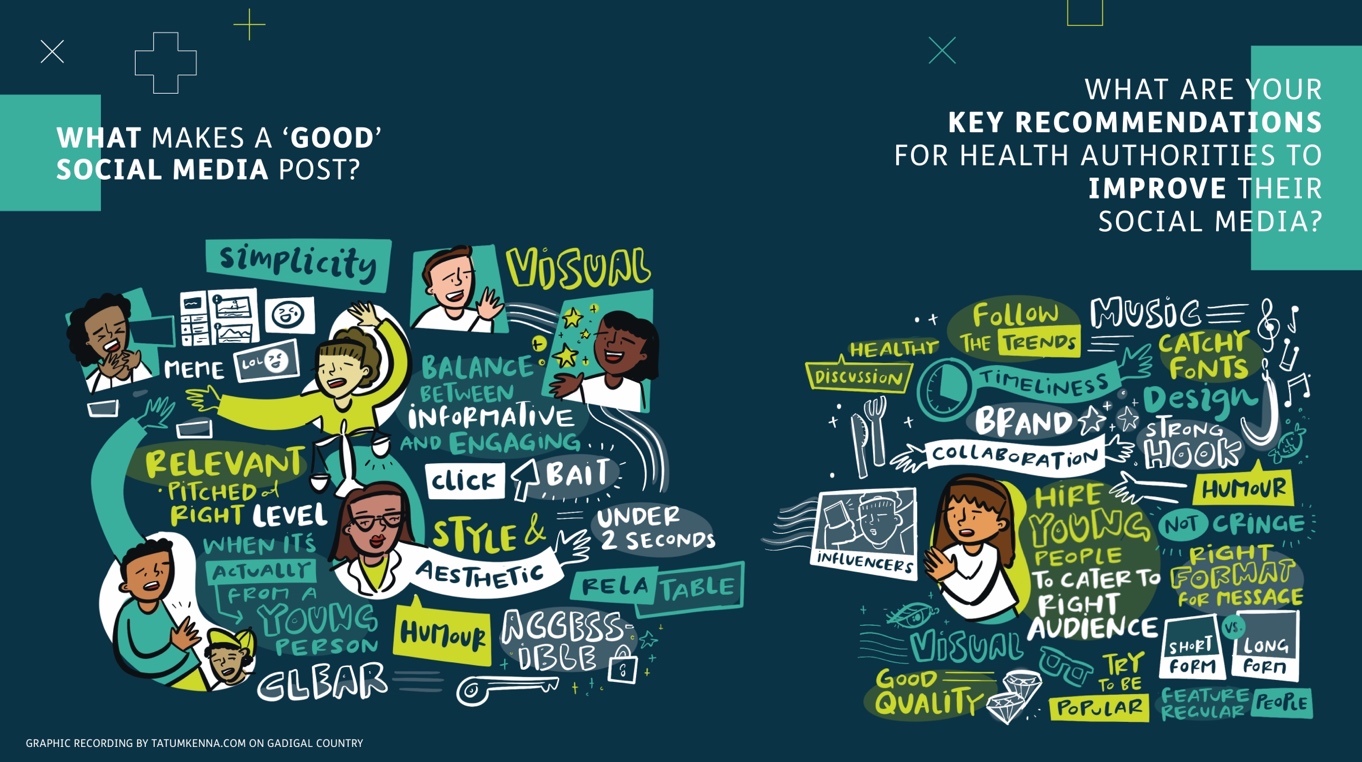
***

*Preferences for official health messaging on social media*

As seen in Figure A, young people preferred simple and clear information that was pitched at the right level. They also liked short content that could capture their interest in a few seconds. As one participant explained: *“Give us bite-sized information instead of being a whole burger.”* Visual appeal was also important, as one participant explained: *“If we’re talking about Instagram or Facebook, the number one thing that catches my attention is like clean, simple graphics with good line spacing in the caption.”* Young people also liked official messaging that was relevant and relatable to them, especially if the message was from a young person: “*I’m more likely to pay attention if there is someone relatable in the post”*.” They also liked health information shared in humorously or by memes and liked click-bait: *“You need some kind of click-bait first, then you have to incorporate a story throughout the post and put a link to more information elsewhere.”*

Young people explained they rarely engaged with health information publicly on social media. There were some exceptions; during emergencies (e.g. COVID-19 pandemic), if information was topical for young people (e.g. mental health awareness) or if sharing that type of content was trendy. Having a call to action encouraging sharing of the message would also increase the chance of them engaging, especially if there was an incentive. One participant explained *“Giveaways regarding health would get me to engage. So for mental health awareness month, give away free counselling sessions to people who engage.”*

Young people explained that seeing other people engaging with social media content would encourage them to read the information. This was especially if it was shared by a friend, with one participating explaining: *“When a friend shares a post, it has a sense of credibility because it's like it they’re the ones saying it. They’re sharing it because they think it’s important, so you know it is important. So it makes you kind of think, ohh I should read this.”* Young people were also drawn to popular posts with lots of likes and comments or trending posts that frequently reappeared on their feeds. Some participants were interested in discourse or “drama” in the comments, with one participant explaining: *“I tend to be engaged with posts with lots of comments…maybe not for the right reasons. Especially with health posts, there’s a lot of controversial and absurd comments underneath. I used to engage with the posts and argue with the anti-vaxxers in the comments”.*

**Box A**: Illustrative quotes for initial concepts from Workshop 1

| 1. **Hire young people**   *“If we are talking about social media, I would rather a young person who actually knows how to communicate to other young people and do good graphic design.”*  *“If they're targeting young people, they should hire young people around of our age to make the posts so it is always relevant to us”*   1. **Feature regular young people**   *“I’m more likely to pay attention if there is someone relatable in the post”*   1. **Create clear and simple messages**   *“Social media is not a good place for sharing long information, it’s not appropriate for the platform. That’s better for an article not Instagram or TikTok”*  *“Give us bite-sized information instead of being a whole burger*  *“Don’t just reshare those medical posters with complicated words that take minutes to read”*   1. **Include easy call to action**   *“I need more than just “get vaccinated”, tell me exactly what steps I need to take to get it.”*  *“It’s good when a post says “Please share to let your close ones know this is how to keep themselves safe”, then I’ll think about it more because it’s got a call to action.”*   1. **Make socially and culturally relevant content**   *“The problem is health authorities aren’t popular with young people in the first place. So you have to get that first before anything else, otherwise you can’t reach the young people.”*  *“It’s important to take into account the seasonality of the events.”*   1. **Create visual-first posts**   *“Honestly, I’d automatically be more interested in the public health agencies if they had good graphic design.”*  *“If we’re talking about Instagram or Facebook, the number one thing that catches my attention is like clean, simple graphics with good line spacing in the caption.”*  *“Avoid being too repetitive the design. Like I don’t want to see “same design. Same design, same design”. I feel like repetitive design is so awkward, monotonous and boring.”*   1. **Capture attention with hooks and clickbait**   *“That's not how social media marketing works. Social media is to attract you to the information, then the link is for when you’re actually into the information, then you go and discover more from there.”*  *“Need a strong hook since health content can be boring.”*  *“You need some kind of click-bait first, then you have to incorporate a story throughout the post and put a link in the description or bio.”*   1. **Use humour, trends, memes**   *“The best way to reach our generation on social media is to go along with the trends while still highlighting the main information that the government wants to get across”*  *“Stay away from cringey stuff. It’s looked down upon. Some trends are viral because it’s bad. That’s not what you want to do.”*  *“Humour is a good way to be engaging. But it needs to be appropriate. Not everyone likes it so don’t over-do it. Like humour is not necessary to explain the difference between COVID and the flu but the funny TikTok video about coming into work sick makes is a good example.”*   1. **Collaborate with influencers**   *“If health information is shared with someone I know or an influencer than I’m more likely to notice the message.”*  *“Like when you think government, you think it's formal, there’s a rigidity to it. It’s inaccessible to a lot of people so collaborating with other organisations or influencers makes it more accessible and gives it a wider audience so it becomes more familiar.”*  *“You need pick the right influencer to collab with so it’s natural. Otherwise it will come across as disingenuous. Social media is about being genuine... or at least coming across as genuine.”*   1. **Make right content for platform**   *“Understand the format that you need for the platform. Post infographics if you're on Instagram. If you're on Twitter and you might write a few sentences, but it's a short form content sharing platform”*  *“Social media is about audio and visual, so both need to be well produced but not too well produced”*  *“Some of the posts that are too polished come across like an ad so I would just scroll past.”*     1. **Balance informative and engaging content**   *“I’m looking for a balance of professional and Zoomer dopamine hit that we need, that’s what social media is for. If I want something serious, I would go for a proper article.”*  *“I feel like you can have two different types of content, like one cater towards more like young audience being more like meme or like trendy format and then the other one being somewhat more informative.”*  *“Well when the younger generation are on social media, they don’t expect or want to see health professionals being interviewed about health issues. We want to find entertainment. That type of content is considered heavy.”*   1. **Involve the public more**   *“ I like when they [public health agencies] respond to comments in a tongue-in-cheek way and shut down the misinformation trolls.”*  *“There is such a large population who have distrust for the government so they it’s good to have healthy and open discussions. Like social media is supposed to be a place where the public can have these discussions with those officials so they can understand and learn about what they do.”* |
| --- |
|  |
|  |

**Workshop 2 Findings**

The stakeholders discussed the feasibility of the ideas raised by the young people, including during times of emergencies during Workshop 2 (Table A).

*Common barriers and enablers*

Stakeholders generally agreed with many of the ideas raised by the young people in previous workshops but outlined barriers and restrictions involved in creating social media content for public health agencies. Barriers included time and cost as well as organisational considerations like approvals, entrenched ways of working and reputational risk considerations for public health agencies: *“Small risks we are willing to take. Bigger risks we steer clear from being a government organisation because can get backlash and lose trust.”*

On the contrary, enablers to enacting these ideas were that social media staff were generally younger in age, experts in social media marketing and aware of ways to increase engagement based on previous experience. For example, one stakeholder explained: *“You will generally get a younger group of people working in social media roles, like in my team we have a 22 year old. They are always on top of the trends. They usually the ones creating content. That’s the nature of social media, you tend to get younger people entering the space.”*

*Considerations during emergencies*

The stakeholders also reported the relevant considerations needed in emergencies for each recommendation. Some recommendations were feasible during emergencies (e.g. pitching message and call to action at right level, capturing attention with hooks making right format and message for the platform). Many recommendations were less feasible due to the limited timeframes and focus on immediate message dissemination: *“Don’t underestimate the time it takes to make content. There's a reason influencers charge a lot for their content and it's cause it takes a lot of time and a lot of thought. And that’s why emergency content is very difficult because you don't have the time.”*

Stakeholders explained that involving or collaborating with external partners like young people or influencers would be difficult in emergencies as there is limited time to identify, train or organise the collaborators. However, enablers identified to these issues include embedding young people in the system (e.g. as interns, or via regularly scheduled focus groups) before emergencies, maintaining relationships with past collaborators and developing a registry of vetted influencers for proactive collaborations. For example, one stakeholder explained: *“You want seasoned people in a crisis. You can't afford to spend much time coaching people but that's part of the preparedness - if you have young people part of the team, they will be ready to go when the time comes.”*

**Table A**: Barriers and enablers identified by stakeholders to young people’s ideas from Workshop 2

| Idea | Barrier | Enabler | Updated recommendation and explanation | Consideration during emergencies |
| --- | --- | --- | --- | --- |
| Hire young people | - age discrimination policies - limited positions funded - limited time to train new staff  ***Quote:*** *“It would be tricky to hire young people as social media officers because you need to be independent and have good judgment because you’re often on shift by yourself.”* | - school/university internships – staff often young anyway - involve in content creation  ***Quote:*** *“For the social team, it's pretty young team as it is, I think mid to late 20s.”* | “**Involve young people in content creation**" (e.g. interns, focus groups, youth panel, collaborations with young content creators)  ***Quote:*** *“Cadetships for people coming out of school or in university as an internship. In doing that you upskill them into the workforce but you also have the benefit of leveraging them for the demographic you’re trying to target.”* | Less feasible during emergencies due to limited time to train, organise & sense check.  Embedding young people in system prior enables this.  ***Quote:*** *“You want seasoned people in a crisis. You can't afford to spend much time coaching people but that's part of the preparedness - if you have young people part of the team, they will be ready to go when the time comes”* |
| Collaborate with influencers and real people | -cost barriers  -limited time to organise  - reputational risk concerns  ***Quote:*** *“First is, you'd have to do a real deep dive into their history to see if anything controversial has ever been done or said. They still could do something controversial down the track so it’s risky. Government entities do not like risk in that way.”* | -talented at communicating to their audience  -practiced with other communities (e.g. Aboriginal and Torres Strait Islander communities)  ***Quote****: “We do yarn ups for the Aboriginal community and create content with the community members. We know they respond better to messages from their own community.”* | **“Collaborate with influencers and real people" (e.g. create registry of vetted talent, collaborate for proactive campaigns, carving out budget for influencers and real people)**  ***Quote****: “The recommendation is that they have a split in those budgets between influencers and then like real people. While an influence is great because they have a good following and they have a, as the name suggests, influence on their audience. If they want the relatable piece that's not normally relatable because they're aspirational.”* | Less feasible during emergencies due to limited time to scope and organise collaborations.  Maintaining relationships and developing registry of vetted collaborators enables this.  ***Quote:*** *“You’d lose some of the aspects of being able to sense check with the youth panel or with a be able to bring a influencer on board.”* |
| Pitch message and call to action at right level | -Difficult to simplify complex medical concepts  -Tailoring requires more work  ***Quote****: “It’s tricky for us because we are working with complex medical information, it has to be accurate and because it’s medical information it needs to be checked by actual doctors because you don't want to run that risk with such a large audience.”* | -include a generic call to action already | **“Pitch message and call to action at right level”**  **(**e.g. reducing complex terms, sense-checking with young person, breaking down call to action into actionable steps)  ***Quote: “****We can maybe strip language down a bit. It’s hard because there are lots of tricky words in health”* | Feasible with little/no extra considerations. |
| Make socially and culturally relevant content | -Requires more time and effort to research | -Value empathy and inclusivity | “**Consider social and cultural context”** (e.g. aligning content to youth events, researching Google trends, sense checking with young person)  ***Quote: “****I'm obviously not going to be across all of the different social issues and cultural norms and what's OK in one culture and not OK in another culture. Think that's where you need to do some research, but also having different like advisory panels would be helpful in those situations. “* | Less feasible during emergencies due to limited time and focus on immediate message sharing.  ***Quote:*** *“Generally in an emergency we will revert back to very plain and simple directing messaging.”* |
| Create visual-first content | -Requires more time and effort to create visual-first content  -Visually heavy content looks like ad  -text-heavy images get high social media engagement sometimes  ***Quote:*** *“The issue is with Facebook and Meta, if you pay for a sponsored post, and the tile has limited text, it looks a lot like advertising, not health information.”* | -Strong visual content gets high engagement too | “**Prioritise visuals”**  (e.g. less text in image, more in caption or link)  ***Quote****: “Not putting text in the graphic is a change for us because we are used to developing fact sheets that are all text and less images. It’s definitely possible but is about re-education.”* | Generally feasible during emergencies but more information needs to be shared. |
| Capture attention with clickbait and hooks | - “clickbait specifically is a negative term  -Platforms do not want users to leave site  ***Quote:*** *“I hate clickbait. It takes up so much time and could have been answered in the post.”* | -Already aim to use hook in content | “**Capture attention with a strong hook”**  (e.g. attention-grabbing image)  ***Quote:*** *“Hook is probably more appropriate, click-bait is a negative term for us”.* | Feasible with little/no extra considerations.  May be less relevant during emergency due to heightened anxiety and interest around topic. |
| Use humour, trends and memes | -Requires more time and effort to research  -Risky if executed poorly  - approval barriers  -Difficult to use humour about most health topics  -Unable to use licensed sounds/music/characters  ***Quote:*** “*There’s this meme of a drag queen backstage looking defeated and the tagline is “Government employees finally getting their meme approved three weeks after it's trending”. I know that feeling so much.”* | -Can use humour around the health issue, rather than topic itself  -Trendy content receives highest engagement  ***Quote:*** *“I got a lot of “No’s” getting some more trendy posts across the line. They often end up being the most engaged with though.”* | **“Use humour, trends and memes”** (e.g. starting with safe and easy trends, collaborating with talent to execute trends, developing trust and streamlined approvals)  ***Quote:*** *“A lot of our issues are at the top. If people at the top aren’t willing to approve, then we can’t do much. But we can build trust with our executive and show them how it works. Then they trust us more.”* | Less feasible during emergencies; limited time and focus is on immediate message sharing.  Difficult to use humour about serious health emergency.  ***Quote:*** *“It's harder to be funny when there is a serious emergency”* |
| Make right format and message for platform | -Dependent on talent (e.g. health professional rarely be suited for TikTok content) | -Over edited and over produced content does not get engagement  - different audiences on different platforms  ***Quote****: “I find that really interesting that they didn’t like the overproducing thing, because we've just been having those discussions in-house now and moving to more low-fi content”* | “**Make the right content for the platform**”  (e.g. research platform norms, select platform presence strategically based on resources)  ***Quote:*** *“We are now trying to be fun on socials regardless of the message is. Those overly staged images just don’t work. They look like an ad. We want to create content your friends would.”* | Feasible with little/no extra considerations |
| Balance informative and engaging content | -Requires more strategic organisation of information  -Difficult to make some more dry and technical health issues engaging  ***Quote:*** *“We've got strict brand guidelines. So we can't just throw anything on a tile or in a video. But we do really try to have a mix of illustrative and photographic images. We try to be as diverse as possible.”* | -Certain health topics are more engaging than others | “**Balance informative and engaging content”**  (e.g. experiment with new formats, sharing range of content including educational, funny, video and infographic posts)  ***Quote****: “We're definitely working towards more of a better balance. Often the “shit posting” stuff is the most shareable.”* | Less feasible during emergencies; limited time and focus is on immediate message sharing.  May be less relevant during emergency due to heightened anxiety and interest around topic. |
| Involve the public more | -Requires more time and effort  -Approval barriers  -Reputational risk barriers to  “shut down” trolls  ***Quote****: “I get that people like to eat their popcorn and watch the drama in comments. But we can’t just respond and stir the pot. We can try to encourage conversation and questioning.”* | -Community engagement is increasingly more recognised for building trust with audience  -helps algorithm performance on the platform  -Have a list of previously approved responses  ***Quote****:* “*Community management is one of the best ways to build trust with your audience. You can directly combat misinformation by responding to things. It helps with the algorithm. When Facebook sees you engaging and then more people engaging with you, it's showing your post to more people, so you're getting that reach. That benefits you in the long run with the rest of your content, because Facebook's picking up on that and you’re building on even more momentum. So it's just gonna help you just get your message out. Not just on that post, but then also subsequent posts from there. “* | **“Engage more with the public”**  (e.g. carving out time for community management, involving health professional in role, increasing list of preapproved comments, allowing for public submissions of content, being honest with audience about capacity)  ***Quote****: “I find it helps to remind people that “hey, there’s no bots here, we are real people just answering the same things over again” so they understand why the answers are similar. Like being reassuring to the audience in that way.”* | Less feasible during emergencies; limited time and focus is on one-way message dissemination.  However, it is very important for audience trust since there is more anxiety/questions.  ***Quote:*** *“During COVID it was very hands on deck. We could access health advisors more quickly. It would be nice to incorporate that process into BAU process.”* |

**Workshop 3 Findings**

The recommendations were finalised by the young people with illustrative examples during Workshop 3 with consideration to the feasibility concerns outlined by the stakeholders in the previous workshop (Box B).

**Box B:** Illustrative quotes and links to youth approved examples for final recommendations from Workshop 3

| 1. **Involve young people**   *“They should start competitions where they encourage young people to make an informative video on some health issue for social media. And maybe there would be a financial incentive.”*  *“I would definitely want to see more internships. I’m a science student and I would love to be an intern but rarely see them advertised. They could do all types of internships on IT, marketing, social media and health and could advertise them at career fairs.”*  Youth approved examples include [links]:  <https://www.tiktok.com/@nswhealth/video/7130896494214778113>   1. **Pitch at right level**   *“If it’s a video post, show the action so we can remember”*  *“Young people like concerts and events so use those trending concerts to get information across”*  *“Try to understand the pulse of the people you’re targeting”*  *“I wouldn’t even always read the caption on TikTok, so even include a call to action to read the caption for more information”*  Youth approved examples include: [links]  <https://www.instagram.com/p/C3i4hphrkpl/?hl=en>  <https://www.instagram.com/p/C3TfSUVNsIF>   1. **Capture attention fast**   *“Strong hooks in the beginning of a health post would keep me interested, even during the boring stats part.”*  *“If something conveys a sense of urgency and danger, that will make me want to engage”*  *“When I see a wall of text, I’m turned off, it’s intimating and I’m like ohh I'm not reading this essay. So I would keep scrolling.”*  *“I think the post on social media should be funny and engaging, but then the link should have more information and be serious. If it's a serious post straight away, I think a lot of people will scroll past it because they don't want to like deal with that as well. But if they say something funny then they are more likely to stop and engage.”*  Youth approved examples include [links]:  <https://www.youtube.com/shorts/PjXDEwW0Tp4>  <https://www.instagram.com/p/C3rkuHmRbNJ/?hl=en>  <https://www.instagram.com/p/Cx56fZ2RZi6/?igshid=NzZhOTFlYzFmZQ%3D%3D&img_index=1>  <https://www.instagram.com/p/C3T-Eg0MGcm/?hl=en>  <https://www.instagram.com/p/CxxFl9hulj_/?igshid=MzRlODBiNWFlZA%3D%3D&img_index=1>  <https://www.instagram.com/p/C3TKkLMtmW7/>   1. **Use current social media marketing**   *“If you're doing a trend or meme, just do it well. I know there is a meme circulating about how as soon as an organisation or company does a meme, it instantly kills the meme.”*  *“It’s a trend to post animals on the internet and it’s pretty safe.”*  *“You need to know the norms of the platform, otherwise the post will just feel off.”*  *“It’s important to use the influencers well. Like make it more authentic, otherwise everyone thinks they are selling out or they got paid to do it. If you’re gonna work with an influencer, give them creative control because we can tell when it’s a script.”*  Youth approved examples include [links]:  <https://www.tiktok.com/@millie/video/7106055331435908354?lang=en>  <https://www.tiktok.com/@dr_idz/video/7252742262461861146?_r=1&_t=8gNuh4iHKsc>  <https://www.instagram.com/p/C30tJb-B1XS/?hl=en>  <https://www.instagram.com/p/Cx6ZOcoBd81/?igshid=MTc4MmM1YmI2Ng%3D%3D>  <https://www.tiktok.com/@thedailyaus/video/7335704636239760658>   1. **Engaging more with the public**   *“I would love it if they responded to DMs. It humanises them and I would show my friends.”*  *“It makes them seem less like an entity and more like a real person.”*  *“You could start a trend, like if you get vaccinated and post it, you could get a prize.”* |
| --- |
|  |
|  |
